# Solid pseudopapillary neoplasm of the pancreas: a rare tumor case report

**DOI:** 10.1097/RC9.0000000000000903

**Published:** 2026-08-21

**Authors:** Ana C. Moreira, Daniela Lira, Maria Costa, Maria Reigota, Filipe Ribeiro, Joana Noronha

**Affiliations:** Department of General Surgery, Unidade Local de Saúde da Região de Aveiro, Aveiro, Portugal

**Keywords:** case report, minimally invasive surgery, pancreas, solid pseudopapillary neoplasm

## Abstract

**Introduction:**

Solid pseudopapillary neoplasm (SPN) of the pancreas is a rare tumor, accounting for about 0.3–2.7% of pancreatic neoplasms. It mainly affects young women aged 20–40 years and usually presents as an asymptomatic abdominal mass. Although typically indolent, approximately 15% exhibit malignant potential. Complete surgical resection is curative in most cases.

**Presentation of the case:**

A 28-year-old woman was referred for a general surgery consultation due to a suspected adrenal gland mass that had been monitored in her country of origin. The patient was asymptomatic, with normal laboratory results and negative tumor markers. Due to an allergy to iodinated contrast, magnetic resonance imaging was performed, confirming findings compatible with an encapsulated pancreatic lesion measuring 112 × 86 × 80 mm. Elective laparoscopic surgery revealed a well-defined mass in contact with the pancreatic tail that was completely excised without complications. Histopathological examination confirmed a solid pseudopapillary neoplasm, with strong β-catenin staining and negative surgical margins. The patient remains asymptomatic under regular follow-up, with no recurrence to date.

**Discussion:**

SPN is a low-grade malignant tumor with an excellent prognosis after complete resection. Diagnosis may be challenging due to overlap with other pancreatic masses, particularly in contrast-restricted studies, and confirmation via β-catenin immunohistochemistry is pivotal. Surgical excision is both diagnostic and therapeutic.

**Conclusion:**

Although rare, SPN should be considered in young women with pancreatic masses. Early recognition and complete surgical removal offer curative outcomes and excellent long-term survival. Minimally invasive, parenchyma-sparing techniques should be considered safe and effective options, particularly in young patients.

## Introduction

Solid pseudopapillary neoplasm (SPN) of the pancreas, previously known as “Frantz” tumor, is a rare entity that accounts for about 0.3–2.7% of all pancreatic cancers^[^1^]^ and was first described by Virginia Kneel and Frantz in 1959^[^2^]^. Originally considered benign or a borderline lesion, the World Health Organization (WHO) reclassified it in 2010 as a low-grade malignant entity^[^3,4^]^. In more than 90% of SPN cases, specific somatic mutations in CTNNB1 exon 3 have been found, which is a gene that encodes β-catenin. Activation of the Wnt/β-catenin pathway occurs, leading to accumulation of β-catenin and loss of membrane expression of E-cadherin^[^5^]^. As a result, immunohistochemistry shows loss of positivity for E-cadherin and positivity for β-catenin, vimentin, synaptophysin, alpha-1-antitrypsin, CD10, CD117, and progesterone receptor, although none are specific for a particular cell line^[^6^]^. The majority of patients have localized disease; however, 10-15% of SPNs present with invasion of adjacent organs or distant metastases^[^7^]^.

HIGHLIGHTS

- Solid pseudopapillary neoplasm of the pancreas predominantly affects young women and presents indolently.
- Diagnosis relies on histopathology and immuno-histochemistry.
- Minimally invasive surgery is organ-sparing and yields similar long-term outcomes.
- Most patients remain disease-free after complete resection.
- Recognition of solid pseudopapillary neoplasm of the pancreas is crucial for curative management.

SPN usually occurs in young women (20–40 years old), although it can appear in individuals of any age or gender^[^8^]^. It predominantly manifests as an abdominal mass that may cause pain, distension, or other symptoms due to tumor compression; nonetheless, patients may be asymptomatic. The treatment for SPN is surgical resection with negative margins, which is curative for most patients^[^1,3,4,7,8^]^.

The objective of this publication is to present this rare entity and to showcase a minimally invasive treatment. This case report has been prepared in line with the SCARE checklist^[^9^]^.

## Presentation of the case

A 28-year-old African female with a personal history of iodinated contrast allergy and no known comorbidities was referred to a general surgery appointment for a suspected mass in the left adrenal gland. The patient was asymptomatic, and the lesion was identified on an abdominal ultrasound performed in Africa 1 year prior to the appointment, following a traffic accident. On physical examination, an abdominal mass was palpable in the left flank and periumbilical area. The mass was non-tender, non-pulsatile, firm, and immobile. A review of systems was otherwise unremarkable. There was no relevant social or family history.

**Figure 1.**
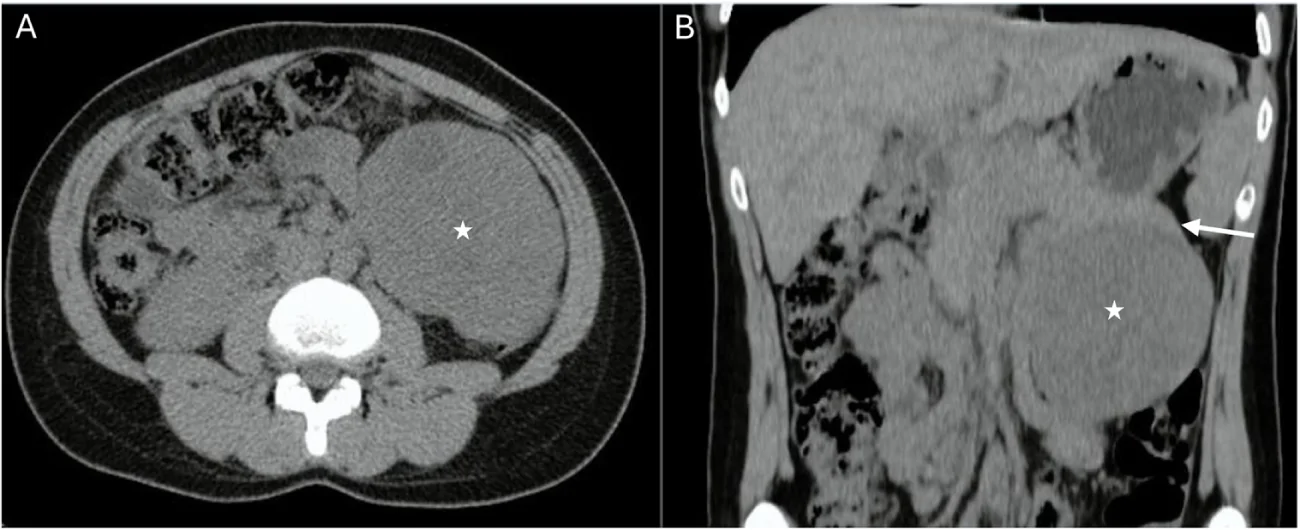
CT scan of the abdomen – axial cut (A) and coronal cut (B) – showing a well-circumscribed mass (identified by a star) without a clear cleavage plane with the pancreatic tail (identified by an arrow).

**Figure 2.**
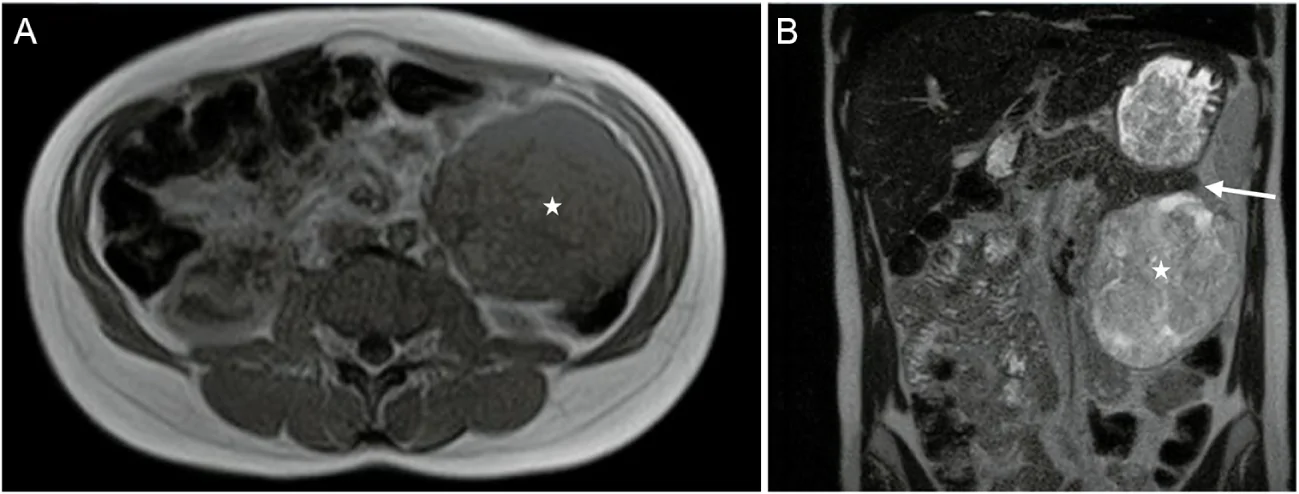
MRI of the abdomen – axial cut, T1 without contrast (A) and coronal cut, T2 with contrast (B) confirming the solid nature of the large lesion with necrotic areas (identified by a star) and dependence of the pancreatic tall (identified by an arrow).

Blood tests were normal, and tumor markers were negative. A computed tomography (CT) scan of the abdomen and pelvis showed a large, well-circumscribed, expansive, non-calcified lesion with predominantly soft-tissue density and slight heterogeneity, measuring 104 × 84 mm in its axial dimensions. The mass did not appear to originate from the left adrenal gland and had no clear plane of cleavage between the mass and the pancreatic tail (Fig. 1). For better characterization, and due to an iodinated contrast allergy, abdominal magnetic resonance imaging (MRI) was performed. The MRI displayed a well-defined, heterogeneous mass with predominantly low signal intensity on T1 and high signal intensity on T2. The lesion was predominantly solid and in contact with the pancreatic tail, with some necrotic areas (Fig. 2). The measured size was 112 × 86 × 80 mm. In both exams, no lymphadenopathy, free fluid, or metastatic implants were observed.

The patient underwent an exploratory laparoscopy, and a large, solid mass with a well-developed capsule, dependent on the tail of the pancreas, was discovered. No ascites, metastatic implants, or carcinomatosis were observed. Subsequently, an enucleation of the mass was performed, allowing for organ preservation (R0 resection). The specimen was placed in an organ retrieval bag and removed through a Pfannenstiel incision. A multichannel drain was placed and directed toward the surgical site. The surgery was uneventful.

Postoperatively, this patient developed a grade A pancreatic fistula, which resolved after 4 days of conservative management (Clavien–Dindo I). The drain was removed on the fifth post-operative day, and the patient was discharged on the sixth day.

**Figure 3.**
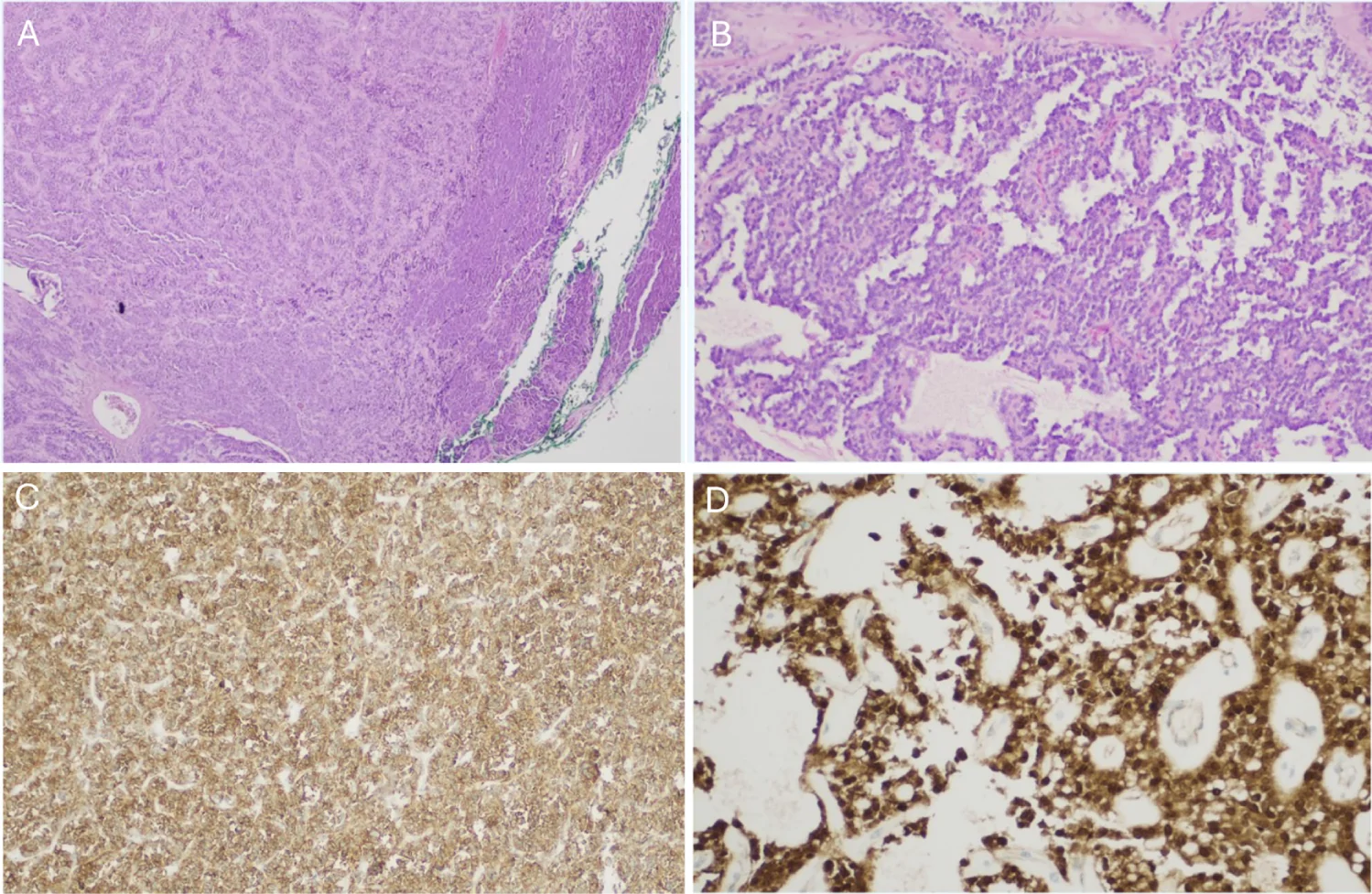
Histopathologic examination with hematoxylin and eosin staining showing papillary morphology (A and B) in relation to the pancreatic parenchyma (A). Immunohistochemical analysis showing tumor cells staining positively for synaptophysin (C) and β-catenin (D).

Histopathology results confirmed complete resection of the lesion, and gross examination of the specimen revealed a large, slightly lobulated, solitary mass measuring 145 × 95 × 75 mm and weighing 408 g. The cross-sectional appearance was solid, with cavitated and congested areas filled with serous material in the central region of the nodule and dense tissue at the periphery. Microscopically, the fragments showed a pseudopapillary morphology with well-defined fibrovascular cores (Fig. 3). The cells displayed cuboidal to oval morphology with centrally located nuclei that were slightly hyperchromatic. Areas of hyalinization and focal cystic degeneration were present, and no atypia or mitotic activity was found. Immunohistochemically, the cells showed heterogeneous positivity for CAM5.2 and focal expression of synaptophysin (Fig. 3), with no immunoreactivity for chromogranin A, PAX8, or Bcl10. There was diffuse and strong nuclear staining with β-catenin (Fig. 3). Margins were negative, and the proliferative index was assessed as below 5% (Ki-67 < 5%). These findings confirmed the diagnosis of a SPN of the pancreas.

On follow-up, the patient remains asymptomatic, and a CT scan performed 1 year after surgery showed no signs of recurrence.

## Discussion

SPN of the pancreas is a rare tumor that predominantly affects young women, particularly in the second to fourth decades of life, although it can also be seen, albeit rarely, in men and children^[^10^]^. The female predominance is marked, with a reported ratio of approximately 10:1^[^11^]^. A higher incidence among Asian and African populations has been described^[^12^]^. This case is representative of this epidemiological pattern, emphasizing the importance of considering SPN in young female patients presenting with pancreatic masses, even when initially mistaken for an extra-pancreatic lesion.

First described by Frantz in 1959^[^2^]^, the tumor has since been recognized by WHO as a distinct entity—SPN – with characteristic morphological and immunohistochemical features^[^3,4^]^. In terms of histopathology, SPNs display characteristic features: well-circumscribed, soft-to-firm masses with solid and cystic areas, often encapsulated, and a combination of solid and pseudopapillary structures formed by uniform epithelial cells arranged around fibrovascular cores. Areas of hemorrhage, cystic degeneration, hyalinization, or necrotic foci can also be found^[^13^]^. The diagnostic immunoprofile of SPNs is essential for distinction from neuroendocrine tumors and other pancreatic tumors and, in this case, positivity for β-catenin and negativity for chromogranin A, PAX8, and Bcl10 were major factors in diagnosing this tumor. A low Ki-67 index (≤5%) denotes slow tumor growth ^[^5,6,14^]^, which was also present in this case.

Clinically, SPN presents with nonspecific symptoms, and incidental detection is becoming more common with the widespread use of imaging. The most common symptoms are abdominal pain, nausea, fullness, or a palpable mass. SPN can grow significantly before causing symptoms, with reported average tumor sizes ranging from 8 to 10 cm at diagnosis^[^5,6,15,16^]^. In this case, the patient was asymptomatic, and the tumor was larger than is usually described.

There are no specific blood investigations or tumor markers, and various imaging techniques can be used in diagnosis. SPNs present as well-circumscribed, encapsulated masses with mixed solid and cystic components. On CT, they manifest as heterogeneous lesions with peripheral enhancement corresponding to the fibrous pseudocapsule^[^17^]^. Notably, the patient’s allergy to iodinated contrast delayed diagnosis and warranted MRI for better tissue characterization without contrast risks. On MRI, SPNs have low signal intensity on T1 and high signal intensity on T2. MRI is superior to CT in detecting certain characteristics such as hemorrhage, the presence of a capsule, or cystic degeneration^[^18^]^. SPNs also show early, heterogeneous, and progressive enhancement compared with adenocarcinomas and endocrine tumors.

Complete surgical excision remains the cornerstone of treatment^[^10,19,20^]^. SPNs are of low-grade malignancy, and more than 95% of patients with SPN have disease limited to the pancreas^[^10^]^. Typically, they are located in the pancreatic body or tail. The operative approach depends on tumor size, location, and relationship to surrounding structures. Conventional pancreatectomy has achieved good results and prognosis; however, enucleation has been increasingly used in the treatment of SPNs^[^21^]^. This approach allows for a parenchyma-sparing alternative and is appropriate for localized disease, given the low risk of lymphatic spread. It has been associated with shorter operative time, less intraoperative blood loss, lower rates of postoperative exocrine insufficiency, and similar overall morbidity^[^21–23^]^. Increasing evidence supports its success via laparoscopic or robotic approaches^[^22,23^]^. In this case, laparoscopic enucleation with subsequent organ preservation and minimal complications is in line with recent trends supporting less extensive surgery. The patient’s symptom-free status and absence of recurrence at 1-year CT follow-up underscore the excellent outcome typical of patients with SPN managed surgically, even when a minimally invasive approach was employed.

The majority of SPNs have localized disease; however, about 10–15% exhibit malignant features, such as invasion of adjacent organs or distant metastases^[^7^]^. Reported recurrence rates are low (approximately 5–15%), particularly when margins are positive or the disease is multifocal, and the 5-year survival rate exceeds 95%, even in the presence of limited metastasis (most commonly hepatic or peritoneal) if they are excised^[^5,7,10,11,16^]^. In these cases, repeat resection or cytoreductive surgery may be considered when an R0 resection or tumor debulking is feasible, although evidence is limited^[^24–27^]^. The role of chemotherapy is not yet well established, with no standard regimen and no consistent proof of benefit, so systemic treatment is generally reserved for unresectable, progressive, or highly selected recurrent disease^[^24,26,28,29^]^. Possible recurrence underscores the need for continued surveillance.

## Conclusion

SPN of the pancreas is a rare, low-grade malignant tumor mostly found in young women. This case illustrates the classic clinical, imaging, and pathological features of SPN. Diagnosis relies on histological and immunohistochemical confirmation, with nuclear β-catenin positivity as a hallmark finding. Complete surgical excision remains the cornerstone of treatment, providing an excellent long-term prognosis. Laparoscopic enucleation enabled complete tumor removal with negative margins while preserving pancreatic parenchyma. Minimally invasive, parenchyma-sparing techniques should be considered safe and effective options, particularly for young patients.

## Data Availability

Not applicable.
